# Association of Waist Circumference and Body Mass Index Deciles Ratio with All-Cause Mortality: Findings from the National Health and Nutrition Examination Survey

**DOI:** 10.3390/nu16070961

**Published:** 2024-03-27

**Authors:** Orna Reges, Tsafnat Test, Dror Dicker, Tomas Karpati

**Affiliations:** 1 Department of Health Systems Management, School of Health Sciences, Ariel University, Ariel 40700, Israel; 2 Branch of Planning and Strategy, Clalit Health Services, Tel Aviv 6209804, Israel; 3 Department of Family Medicine, Faculty of Health Sciences, Ben-Gurion University of the Negev, Beer-Sheva 8410501, Israel; drtestts@clalit.org.il; 4 Clalit Health Services, Southern District, Yerucham 8050315, Israel; 5 Internal Medicine D and Obesity Clinic, Hasharon Hospital, Rabin Medical Center, Petah Tikva 4937211, Israel; 6 Faculty of Medicine, Tel-Aviv University, Tel Aviv 6997801, Israel; 7 Department of Digital Technologies in Medicine, Holon Institute of Technology, Holon 5810201, Israel; tomask@hit.ac.il

**Keywords:** waist circumference, BMI, obesity, mortality

## Abstract

Given the importance and continued interest in finding a simple, accessible, and universal measure which reflects both general and abdominal adiposity, this study tested for an association of the ratio of WC decile to BMI decile (WC-d/BMI-d) with all-cause mortality. Individuals aged 18–79 years who had participated in the National Health and Nutrition Examination Survey (NHANES) during the years 2007 to 2018 were included in the analysis. WC and BMI deciles were defined separately for males and females, while WC-d/BMI-d was calculated for each individual. The association of WC-d/BMI-d with mortality was assessed using logistic models for the total study population, and then again after stratification by sex, ethnicity, morbidity level, and BMI categories. Positive associations between WC-d/BMI-d and mortality were demonstrated for the total study population (adjusted OR = 1.545, 95%CI: 1.369–1.722) and within different sub-groups, including the population with a normal BMI level (adjusted OR = 1.32, 95%CI: 1.13–1.50). WC-d/BMI-d increased with age, with ~40 years representing a critical time point when WC-d surpasses BMI-d, with a sharper incline for males as compared to females. WC-d/BMI-d was significantly associated with all-cause mortality amongst NHANES American adults; thus, measurements of WC and its integration with BMI in this metric should be considered in clinical practice.

## 1. Introduction

Obesity, characterized by abnormal or excessive fat accumulation, is increasing in prevalence worldwide [1]. There are several anthropometric indices which aim to define obesity, of which the most widespread is body mass index (BMI), calculated as the ratio between body mass and height squared [2,3]. This index defines four main categories: Underweight, Normal weight, Overweight, and Obese. The BMI cutoffs for these four categories are the same for males and females, and all ages (above 18 years). The major limitation of BMI lies in its inability to measure fat distribution or visceral fat [4,5,6], which were found to be risk factors for cardiometabolic morbidity [7]. One of the common anthropometric indices used to measure central fat distribution is waist circumference (WC) [4,8]. Unlike BMI, the measurement of WC is less standardized [9] and there are diverse cutoffs by gender and different ethnic groups (e.g., according to the World Health Organization (WHO), for people of European origin, >80 cm for females and >94 cm for males, and for people of Asian ethnicity, >80 for females and >90 for males [10,11]; while according to the American Heart Association (AHA), >88 cm for females and >102 cm for males) [12]. Both indices, BMI and WC, increase with age [13,14], with a greater increase for WC [15], yet changes in WC relative to BMI, especially for a given BMI category, are not fully understood. 

The association of obesity with cardiovascular disease (CVD) and mortality is well established [6,16,17]. Both BMI and WC have been found to be independent risk factors for CVD and mortality [18,19,20,21]. Furthermore, a recently published Consensus Statement from the International Atherosclerosis Society (IAS) and International Chair on Cardiometabolic Risk (ICCR) Working Group on Visceral Obesity argued that BMI alone is not sufficient to assess cardiometabolic risk. This Consensus Statement proposed WC as an additional important measurement [22] and emphasized the importance of considering WC within the specific BMI categories. Indeed, several studies have shown that an increase in WC within each BMI category was associated with increased mortality risk [23,24,25]. Few studies have also recommended using an index which combines BMI and WC in order to assess its association with morbidity and mortality, especially in middle-aged and older populations [26,27,28]. For instance, Liu et al. used the ratio between continuous values of WC and BMI to demonstrate higher risk for mortality associated with this obesity phenotype [26]. A meta-analysis by Jayedi et al. [27] demonstrated a significant positive association of a body shape index (ABSI, defined as WC (cm)/[BMI2/3 × height (cm)1/2]) [29] and all-cause mortality risk. 

Given the importance of and continued interest in finding a simple, accessible, and universal measure which reflects these two obesity-related metrics, the current study suggests using the ratio of WC decile and BMI decile (WC-d/BMI-d). This metric can be used to identify a different phenotype of obesity, specifically the increase in this ratio indicates a change from obesity with general fat distribution to a form with central fat distribution. The objectives of this study are to (1) describe the distribution of the population by obesity level using WC-d/BMI-d; (2) assess the association of this metric with all-cause mortality; and (3) observe changes in WC-d/BMI-d with increasing age, stratified by sex and BMI categories.

## 2. Materials and Methods

### 2.1. Study Design and Participants

De-identified and publicly available data were extracted from the National Health and Nutrition Examination Survey (NHANES), which can be accessed at http://www.cdc.gov/nchs/nhanes/index.htm (accessed on 15 March 2023). NHANES is a cross-sectional representative survey of non-institutionalized citizens in the United States, which collects data every 2 years. The sample for the survey was selected to represent the U.S. population of all ages. For this study, individuals aged 18–79 years who had participated in NHANES during the years 2007 to 2018 were selected. Individuals with missing BMI or WC, individuals with BMI below 18, and pregnant women were excluded from the analysis.

### 2.2. Main Exposure (WC-d/BMI-d) and Covariates

WC and BMI deciles were defined separately for males and females. For each gender, individuals were sorted in ascending order based on their WC values, and the range of values was divided into ten equal deciles. Similarly, deciles were defined for BMI. Once individuals were assigned to their respective WC and BMI deciles, these deciles were encoded numerically from 1 to 10, and the ratio between the encoding for both measures (WC-d/BMI-d) was calculated for each individual. A WC-d/BMI-d equal to 1 indicated that an individual ranked in the same decile for BMI and WC. Ratios greater than 1 suggested that an individual ranked in a higher WC decile relative to their BMI decile, while ratios less than 1 indicated the opposite. 

Demographic measures (age, ethnicity, and smoking category), clinical measures (systolic blood pressure (SBP), diastolic blood pressure (DBP), glucose, cholesterol (low-density lipoproteins (LDL), high-density lipoproteins (HDL), and triglycerides), and chronic diagnoses (diabetes, hypertension, coronary artery disease (CAD), congestive heart failure (CHF), renal disease, and asthma) were selected as possible covariates. Individuals were defined as non-morbid, morbid (having 1 of the above chronic diseases), or multi-morbid (having ≥ 2 of the above chronic diseases).

### 2.3. Outcomes 

The primary outcome of this study was overall mortality. For NHANES participants, mortality status was ascertained through a probabilistic algorithm for record matching with the National Death Index at https://www.cdc.gov/nchs/data-linkage/mortality-public.htm (accessed on 15 March 2023).

### 2.4. Statistical Analysis

The main characteristics for the total study population and populations stratified by sex were described for categorical variables using proportions, and for continuous variables using means with standard deviations (SD) or medians with interquartile ranges (IQR). Differences between males and females were assessed using the Chi-Square test for categorial variables. Differences in continuous variables were evaluated using unpaired *t*-tests for variables with normal distribution or the Mann–Whitney U test for non-normally distributed variables. 

The association of WC-decile/BMI-decile (as a continuous variable) with mortality was assessed using logistic models for the total study population and for populations stratified by sex, ethnicity, morbidity level, and BMI categories. The model for the entire population was adjusted for age, gender, ethnicity, morbidity level, and BMI. An additional model was utilized to account for each chronic disease as an independent variable. Stratified models were adjusted for all covariates except the covariate used for stratification. A forest plot graph was used to describe the odds ratios of the unadjusted and adjusted models.

Linear regression models were used to assess the independent association of age with WC-d/BMI-d for the total study population and populations stratified by sex and BMI categories. The linear model for the total population was adjusted for gender, ethnicity, co-morbidity, and BMI. Stratified models were adjusted for all covariates except the covariate used for stratification. 

All the analyses were performed using R statistical software version 4.0.2. 3 [30]. 

## 3. Results

### 3.1. Descriptive Statistics

Of the 59,842 individuals who participated in NHANES during the years 2007 to 2018, there were 34,198 individuals aged 18–79 years. Of these, 3900 were excluded due to (1) missing BMI or WC data (3001), (2) BMI < 18.5 (570), or (3) pregnancy at the time of inclusion (329). Individuals with missing BMI or WC were more likely to have diabetes (15.7%), CAD (4.8%), and CHF (5.7%), as well as higher all-cause mortality (12.8%), than those included in the analysis. The final study population consisted of 30,298 individuals (mean age: 46.4 ± 17.1 years, 50.3% female). The main characteristics of the study population are described in Table 1. The mean BMI level of the participants was 29.3 ± 6.8 (male: 28.8 ± 6.0; female: 29.8 ± 7.5) and the mean WC was 99.3 ± 16.5 (male: 100.7 ± 16.1; female: 97.8 ± 16.8).

### 3.2. WC-d/BMI-d Distribution

**Figure 1:**
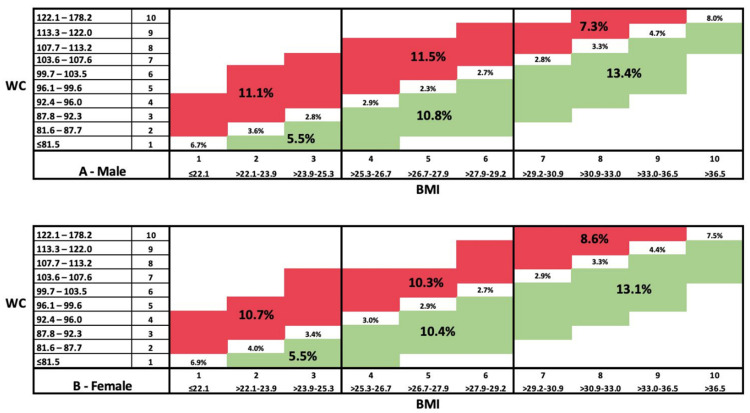
Distribution (%) by body mass index and waist circumference deciles (based on gender deciles). A. Males; B. females. Abbreviations: body mass index (BMI), waist circumference (WC).

Figure 1 (A + B) visualizes the distribution of the study population by WC and BMI deciles. For both males and females, about 40% (39.8% and 41%, respectively) ranked on the same decile of BMI and WC, meaning that they had a WC-d/BMI-d equal to 1 (represented by the white diagonal). About 30% (29.9%, and 29.6%, respectively) were ranked on a higher decile for WC than for BMI, meaning that they had a WC-d/BMI-d greater than 1 (above the diagonal in red). The remaining 30% (29.7% and 29%, respectively) were ranked on a lower decile for WC than BMI, meaning that they had a WC-d/BMI-d less than 1 (below the diagonal in green). 

When looking only at the ~30% of individuals who ranked on a higher decile for WC relative to BMI, this percentage can be further divided into 11.1%, 11.5%, and 7.3% for normal BMI, overweight, and obesity, respectively, in the male population, and 10.7%, 10.3%, and 8.6%, respectively, in the female population. For the ~30% of individuals who ranked lower for WC than BMI, this percentage can be divided into 5.5%, 10.8%, and 13.4% for normal BMI, overweight, and obesity, respectively, for males, and 5.5%, 10.4%, and 13.1%, respectively, for females. 

### 3.3. Association of WC-d/BMI-d with All-Cause Mortality

**Figure 2:**
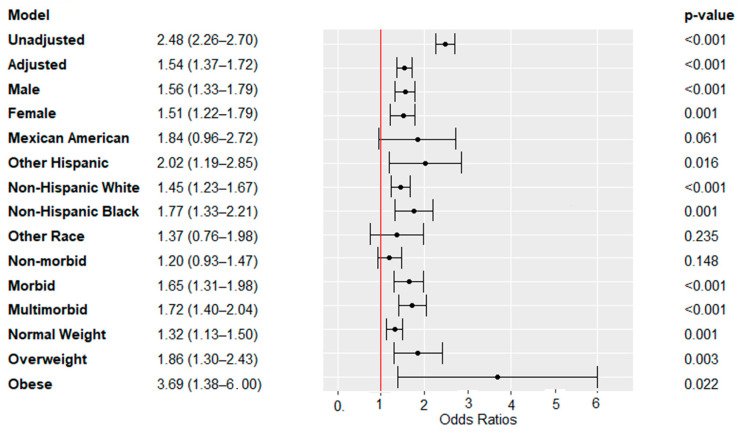
Association of WC-d/BMI-d with all-cause mortality. Dependent variable: all-cause mortality; main exposure: WC-d/BMI-d (continuous scale), calculated separately for males and females. The model for the total population was adjusted for age, gender, ethnicity, co-morbidity, and BMI level. Stratified models were adjusted for all covariates except the covariate used for stratification.

Associations of WC-d/BMI-d with all-cause mortality are described in Figure 2. Among the total population, a higher risk was observed as the ratio between WC-d and BMI-d increased (adjusted OR = 1.545, 95%CI: 1.369–1.722). An additional model which accounted for each chronic disease as an independent variable yielded consistent results (adjusted OR = 1.537; 95%CI: 1.360–1.714).

The significant association was preserved separately for males (adjusted OR = 1.56; 95%CI: 1.33–1.79); females (adjusted OR = 1.51; 95%CI: 1.22–1.79; Non-Hispanic White (adjusted OR = 1.45; 95%CI: 1.23–1.67); Non-Hispanic Black (adjusted OR = 1.77; 95%CI: 1.33–2.21); individuals with one morbidity (adjusted OR = 1.65; 95%CI: 1.31–1.98); individuals with multi-morbidities (adjusted OR = 1.72; 95%CI: 1.40–2.05); individuals with normal BMI levels (adjusted OR = 1.32; 95%CI: 1.13–1.50); individuals with overweight (adjusted OR = 1.87; 95%CI: 1.30–2.43); and individuals with obesity (adjusted OR = 3.69; 95%CI: 1.38–6.00). No significant associations were demonstrated for Mexican Americans (adjusted OR = 1.84; 95%CI: 0.96–2.73); Other Race (adjusted OR = 1.37; 95%CI: 0.76–1.98), or individuals free of chronic disease (adjusted OR = 1.20; 95%CI: 0.93–1.47).

#### Association of Age with WC-d/BMI-d

Multivariable linear regression demonstrated a significant positive association of age with WC-d/BMI-d (0.008, *p* < 0.001).

**Figure 3:**
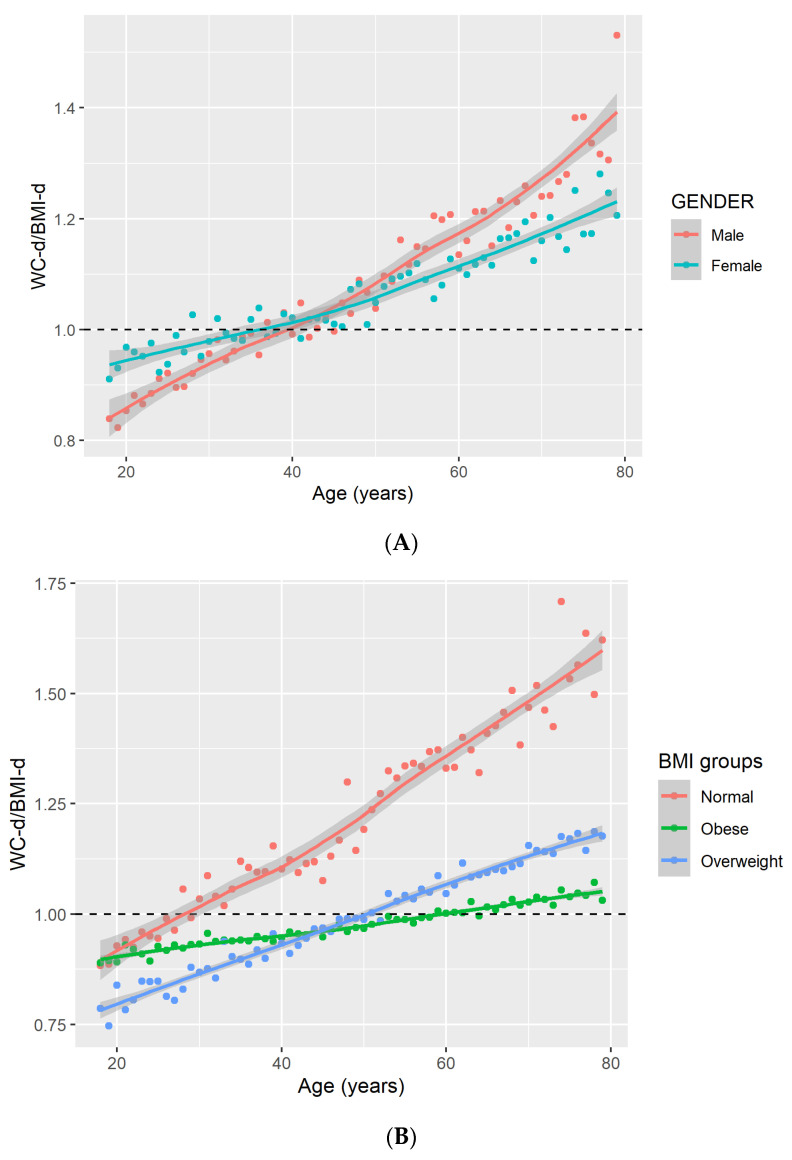
Changes in the ratio of waist circumference-decile to BMI-decile with age by gender (**A**) and by BMI group (**B**).

When stratified by sex (*p* < 0.001 for age × sex), a similar significant increasing trend of WC-d/BMI-d with age was found for males (0.013, *p* < 0.001) and females (0.008, *p* < 0.001), with greater increases for males (Figure 3A). Specifically, around the age of 40 years, this ratio was greater than 1 for both males and females.

Similarly, the association of age with WC-d/BMI-d (*p* < 0.001 for age x BMI categories) was also significant in all of the BMI groups examined (normal weight: 0.010, *p* < 0.001; overweight: 0.006, *p* < 0.001; and obesity: 0.002, *p* < 0.001), with the greatest increases found in individuals with a normal BMI level (Figure 3B). The age at which the ratio became greater than 1 differs in the different BMI categories, with an increase as the BMI category increased: age ~25 in individuals with normal BMI, age ~50 in individuals with over-weight, and ~60 in individuals with obesity. 

The line depicts the overall trend based on locally weighted scatterplot smoothing (LOWESS) and its 95% confidence interval of WC-d/BMI-d. The dots represent individual data points along that trend. 

## 4. Discussion

In this study, amongst adults who had participated in NHANES during the years 2007 to 2018, WC-d/BMI-d was significantly and directly associated with mortality, with increased risk as the ratio between WC-d and BMI-d increased. The significant association was preserved even after adjustment for BMI level, as well as separately for males and females, and within the different BMI sub-groups. About one-third of the population had a higher decile for WC than for BMI (i.e., WC-d/BMI-d greater than 1). Furthermore, WC-d/BMI-d increased with age in both males and females, with age ~40 years representing a critical time point when WC-d surpassed BMI-d. There was a sharper incline for males as compared to females. The association of age with WC-d/BMI-d was also observed separately for BMI sub-groups, with the greatest increases among individuals with normal BMI levels.

The association between BMI and mortality is well established [6,16,17,28,31,32]. WC has also been found to be a strong risk factor for mortality even when adjusting for BMI [23,28,31,33,34] and sometimes even stronger than BMI [32]. Previous studies that considered both WC and BMI either assessed the association of WC with mortality stratified by BMI groups or used different variations of an integrated index of WC and BMI. Studies that stratified results by BMI groups demonstrated varied findings with some evidence for associations between WC and mortality across all BMI groups [23,34], and some showing associations only in specific BMI groups [32,35,36]. Similar to previous studies where WC and BMI were integrated into a single index [26,27], the present study observed an elevated risk associated with an increase in the combined index. A meta-analysis by Jayedi et al. [27] expanded on the significant association between the index ABSI with all-cause mortality by indicating that heterogeneity in potential risk could be attributed to differences between sub-groups based on BMI or pre-existing diseases. Heterogeneity in mortality risk was also demonstrated in this study.

A recent Consensus Statement from the IAS and ICCR Working Group on Visceral Obesity emphasized the importance of considering WC within the specific BMI categories [22]. In the current study, the inclusion of one measure, which contains information about the ratio between WC deciles and BMI deciles, allowed for an analysis of the risk of these combined factors on mortality overall, and also within the different sub-groups such as sex and BMI categories. A significant association of WC-d/BMI-d with mortality was demonstrated separately for all BMI categories. The significant association observed among individuals with a normal BMI level is of particular interest. From a clinical perspective, individuals with a normal BMI level are considered low-risk; however, with the addition of the WC-d/BMI-d index emerges a sub-group, within the normal BMI group, of individuals with increased risk for mortality. The significant association between the increase in WC-d/BMI-d and mortality in the overweight and obese BMI categories is also important to note, as it signifies that this association remained consistent across the different groups. 

Using deciles to segment the population in the current study allowed for a more precise visualization of the distribution of the population based on these two health-related metrics. This visualization enabled the identification of homogenous clusters of individuals at higher risk for mortality (WC-d/BMI-d higher than 1) in the total population and within the different BMI groups. Specifically, about a third of the population had a higher decile for WC than for BMI, which comprised ~11% normal BMI, ~12% overweight, and ~7% individuals with obesity. 

Previous studies demonstrated linear increases in WC with age [13]; furthermore, there is evidence indicating that WC is increasing at a steeper incline than expected considering a given BMI level [22]. In the current study, in both sexes and for all BMI categories, there was an increase in WC-d/BMI-d with age. This increase was steeper among males as compared to females, and in normal BMI as compared to other BMI categories. Integrating WC and BMI into a single metric in the current study facilitates the evaluation and confirmation of the different phenotypes of obesity and how they change with age. 

Less is known about the critical time periods when WC increases relative to BMI. The current study indicates that there may be critical time windows when this ratio changes and that these time windows differ in relation to the different sub-groups. While for the total population there seems to be a change at ~40 years where WC-d increases relative to BMI-d, when looking at individuals in the different BMI categories, the age at which this change happened increased with the increase in BMI category (~25 years for normal, ~50 years for overweight, and ~60 years for obesity). Therefore, follow-up at younger age may be beneficial in identifying individuals with higher risk for mortality based on higher WC-d/BMI-d, especially among individuals with a normal BMI. The differences between the different sub-groups also emphasizes the importance of a more individualized approach to assessing risk using these measures. Longitudinal studies are necessary to refine these specific time windows and more deeply understand their significance.

A strength of this study was the utilization of a simple, accessible, and easily interpretable measure that reflects and combines two crucial obesity-related metrics: BMI and WC. Furthermore, employing deciles to partition the study populations provided a higher level of detail for illustrating the distribution of the population based on WC and BMI. Using deciles also facilitated the application of WC-d/BMI-d in different populations with varying distributions of WC and BMI. Subsequent studies should assess this decile-based metric for additional populations and diverse ethnic groups. Lastly, the large sample size from a well-established US cohort allowed for the performance of stratified analyses and demonstrated the robustness of the positive association between WC-d/BMI-d and mortality. This study also has limitations. First is the observational nature of the study and the potential of residual confounding, even after accounting for potential confounders. Second, 3001 individuals were excluded from the analysis due to missing BMI or WC data. These individuals were more likely to be morbid and had higher all-cause mortality than those included in the analysis, potentially impacting the generalizability of the results. Third, due to the cross-sectional nature of NHANES, measurements of WC and BMI were captured only at a single time point, providing a snapshot rather than facilitating a dynamic understanding of their trajectories. This absence of longitudinal evidence restricts the ability to identify and consider changes in WC and BMI over time. Future studies should prioritize longitudinal study designs, which enable the consideration of the trajectories of WC and BMI.

## 5. Conclusions

WC-d/BMI-d was significantly associated with all-cause mortality in the NHANES American adults. Measurements of WC and its integration with BMI in this metric should be considered in clinical practice. 

## Figures and Tables

**Table 1:** Main characteristics of the study population.

		All Population	Male	Female	*p*-Value
Individuals	n	30,298	15,054	15,244	
Age, years	Mean (SD)	46.3 (17.1)	46.2 (17.3)	46.5 (16.9)	0.071
	Median (IQR)	46.0 (32.0–61.0)	46.0 (31.0–61.0)	46.0 (32.0–61.0)	
Age groups, n (%)	20–39	11,548 (38.1%)	5864 (39.0%)	5684 (37.3%)	0.001
	40–59	10,316 (34.0%)	4980 (33.1%)	5336 (35.0%)	
	60+	8434 (27.8%)	4210 (28.0%)	4224 (27.7%)	
Ethnicity, n (%)	Mexican American	4916 (16.2%)	2437 (16.2%)	2479 (16.3%)	<0.001
	Other Hispanic	3342 (11.0%)	1517 (10.1%)	1825 (12.0%)	
	Non-Hispanic White	11,547 (38.1%)	5857 (38.9%)	5690 (37.3%)	
	Non-Hispanic Black	6678 (22.0%)	3296 (21.9%)	3382 (22.2%)	
	Other Race	3815 (12.6%)	1947 (12.9%)	1868 (12.3%)	
Body mass index (BMI)	Mean (SD)	29.3 (6.8)	28.8 (6.0)	29.8 (7.5)	<0.001
	Median (IQR)	28.1 (24.4–32.8)	27.8 (24.5–31.8)	28.6 (24.2–33.9)	
BMI groups, n (%)	Normal (BMI < 25)	8683 (28.7%)	4209 (28.0%)	4474 (29.4%)	<0.001
	Overweight (25, <30)	9889 (32.6%)	5587 (37.1%)	4302 (28.2%)	
	Obese (≥30)	11,726 (38.7%)	5258 (34.9%)	6468 (42.4%)	
Waist circumference, cm	Mean (SD)	99.3 (16.5)	100.7 (16.1)	97.8 (16.8)	<0.001
	Median (IQR)	97.7 (87.2–109.0)	99.2 (89.5–109.9)	96.0 (85.2–108.0)	
WC-d/BMI-d	Mean (SD)	1.06 (0.39)	1.07 (0.41)	1.05 (0.37)	0.016
	Median (IQR)	1.0 (0.88–1.14)	1.0 (0.88–1.14)	1.0 (0.89–1.14)	
Smoking status, n (%)	Non-smoker	16,737 (55.2%)	6951 (46.2%)	9786 (64.2%)	<0.001
	Past smoker	6568 (21.7%)	4054 (26.9%)	2514 (16.5%)	
	Smoker	6211 (20.5%)	3610 (24.0%)	2601 (17.1%)	
	Missing	782 (2.6%)	439 (2.9%)	343 (2.3%)	
Glucose, mmol/L	Mean (SD)	5.7 (2.2)	5.8 (2.3)	5.6 (2.1)	<0.001
	Median (IQR)	5.2 (4.7–5.8)	5.2 (4.8–5.8)	5.0 (4.7–5.7)	
	Missing (%)	1621 (5.4%)	804 (5.3%)	817 (5.4%)	
Total cholesterol, mmol/L	Mean (SD)	5.0 (1.1)	4.9 (1.1)	5.0 (1.1)	<0.001
	Median (IQR)	4.9 (4.2–5.6)	4.8 (4.1–5.6)	4.9 (4.3–5.7)	
	Missing (%)	1626 (5.4%)	806 (5.4%)	820 (5.4%)	
LDL, mmol/L	Mean (SD)	113.2 (35.4)	113.1 (35.5)	113.4 (35.3)	0.55
	Median (IQR)	110.0 (89.0–134.0)	111.0 (88.0–135.0)	110.0 (89.0–134.0)	
	Missing (%)	16,686 (55.1%)	8365 (55.6%)	8321 (54.6%)	
HDL, mmol/L	Mean (SD)	52.3 (15.8)	47.8 (14.3)	56.7 (16.0)	<0.001
	Median (IQR)	50.0 (41.0–61.0)	45.0 (38.0–55.0)	55.0 (45.0–66.0)	
	Missing (%)	1526 (5.0%)	769 (5.1%)	757 (5.0%)	
Triglycerides, mmol/L	Mean (SD)	1.7 (1.5)	1.9 (1.7)	1.5 (1.3)	<0.001
	Median (IQR)	1.3 (0.9–2.1)	1.5 (1.0–2.3)	1.3 (0.8–1.9)	
	Missing (%)	1639 (5.4%)	810 (5.4%)	829 (5.4%)	
Systolic blood pressure, mm Hg	Mean (SD)	123.3 (17.8)	125.2 (16.6)	121.4 (18.8)	<0.001
	Median (IQR)	120 (112–132)	122.0 (114–134)	118.0 (108–132)	
	Missing (%)	2075 (6.8%)	867 (5.8%)	1208 (7.9%)	
Diastolic blood pressure,	Mean (SD)	70.8 (12.6)	72.2 (12.8)	69.4 (12.2)	<0.001
mm Hg	Median (IQR)	72.0 (64.0–78.0)	72.0 (64.0–80.0)	70.0 (62.0–76.0)	
	Missing (%)	2075 (6.8%)	867 (5.8%)	1208 (7.9%)	
Chronic diseases, n (%)	Diabetics	3660 (12.1%)	1906 (12.7%)	1754 (11.5%)	0.008
	Hypertension	9987 (33.0%)	4906 (32.6%)	5081 (33.3%)	0.277
	CAD	946 (3.1%)	657 (4.4%)	289 (1.9%)	<0.001
	CHF	731 (2.4%)	438 (2.9%)	293 (1.9%)	<0.001
	Renal disease	824 (2.72%)	405 (2.69%)	419 (2.74%)	0.008
	Asthma	4529 (14.9%)	1946 (12.9%)	2583 (16.9%)	<0.001
Co-morbidities, n (%)	Non-morbid	15,753 (52.0%)	7989 (53.1%)	7764 (50.9%)	<0.001
	Morbid	8459 (27.9%)	4037 (26.8%)	4422 (29.0%)	
	Multi-morbid	6086 (20.1%)	3028 (20.1%)	3058 (20.1%)	
Deaths, n (%)		1856 (6.1%)	1115 (7.4%)	741 (4.9%)	<0.001
	Missing	69 (0.2%)	26 (0.2%)	43 (0.3%)	

## Data Availability

The datasets generated and analyzed during for this study are available in the National Health and Nutrition Examination Survey (NHANES), https://www.cdc.gov/nchs/nhanes/ (accessed on 15 March 2023).
